# Enhancing tube feeding method for neurosurgery: the application of improved PICC technique

**DOI:** 10.1186/s40001-024-01729-3

**Published:** 2024-02-17

**Authors:** Huiwen Wu, Yuru Qiu, Yucui Wang, Jiarong Li, Yihong Qiu

**Affiliations:** 1 Department of Neurosurgery, Sun Yat-Sen University Sun Yat-Sen Memorial Hospital, Guangzhou, China; 2 Department of Nursing, Sun Yat-Sen University Sun Yat-Sen Memorial Hospital, Guangzhou, China; 3 Department of Surgery, Sun Yat-Sen University Sun Yat-Sen Memorial Hospital, Guangzhou, China

**Keywords:** PICC, Catheterization, Intracavitary ECG, P wave amplitude change, Catheter tip misplacement rate

## Abstract

**Background and purpose:**

Peripherally inserted central catheter (PICC) used in neurosurgical patients requires changes in patients' head positions. However, such changes can worsen pressure on the brain tissue, lead to sudden acute brain herniation and respiratory arrest, resulting in a higher chance of patient death. This paper addresses the aforementioned problems by introducing a new PICC catheterization method.

**Method:**

In a retrospective study, the records of patients with PICC from April 2020 to April 2023 were reviewed, and they were divided into three groups based on the methods employed. The first group as the conventional group, involved changing patients’ body positions during catheterization. The second group, as the intracavitary electrocardiographic (IECG) group, utilized intracavitary electrocardiographic monitoring and involved changing patients’ body positions during catheterization. The third group as the intracavitary electrocardiographic with improved body positioning (IECG-IBP) group, catheterization was performed with guidance from intracavitary electrocardiographs and without changing the patients’ body positions. The ECG changes among patients undergoing different catheter delivery methods were then compared, as well as the rate of catheter tip misplacement.

**Result:**

The study encompassed a total of 354 cases. Our findings reveal distinct P wave amplitude percentages among the groups: 0% in the conventional group, 88.46% in the IECG group, and 91.78% in the IECG-IBP group. Furthermore, the following catheter tip misplacement rates were recorded: 11.54% for the conventional group, 5.39% for the IECG group, and 5.47% for the IECG-IBP group. Significantly notable differences were observed in these two key indicators between the conventional group and the IECG-IBP group. Notably, the IECG-IBP group demonstrated a more favorable outcome compared to the IECG group.

**Conclusion:**

In patients with neurosurgical diseases, especially those with tracheostomy and nuchal stiffness, the IECG-IBP PICC catheter insertion method can effectively reduce the patient's neck resistance, does not increase the patient's headache and dizziness symptoms, and does not reduce the success of one-time catheterization. Rate and does not increase the incidence of jugular venous ectopia.

## Introduction

During hospitalization, neurosurgery patients, particularly those with conditions such as sudden hemorrhagic stroke or large brain tumors, are often critically ill. Efficient venous access is crucial in such cases. Repeated venipuncture not only causes pain to patients but also increases the workload of nurses [1–4]. To address these challenges, peripherally inserted central catheters (PICC) are widely used in intravenous infusion therapy, reducing the risk of blood and pneumothorax complications [5–7]. Hyperbaric injectable PICCs have gained popularity in neurosurgery as they meet the treatment needs of patients and are preferred over internal jugular vein or subclavian vein catheterization [8]. The implementation of intracavitary electrocardiographic monitoring-guided PICC tip positioning technology has proven effective in reducing the rate of catheter tip ectopia [9–14]. Neurosurgery patients with neck stiffness face limitations in head and neck movement, while those with severe brain tissue compression are at a higher risk of brain herniation and respiratory arrest upon significant head movements. Therefore, this paper explores an improved catheter delivery method aimed at reducing the incidence of catheter tip dislocation during PICC catheterization and minimizing the factors that contribute to intracranial hypertension.

## Methods

### Participants

**Fig. 1:**
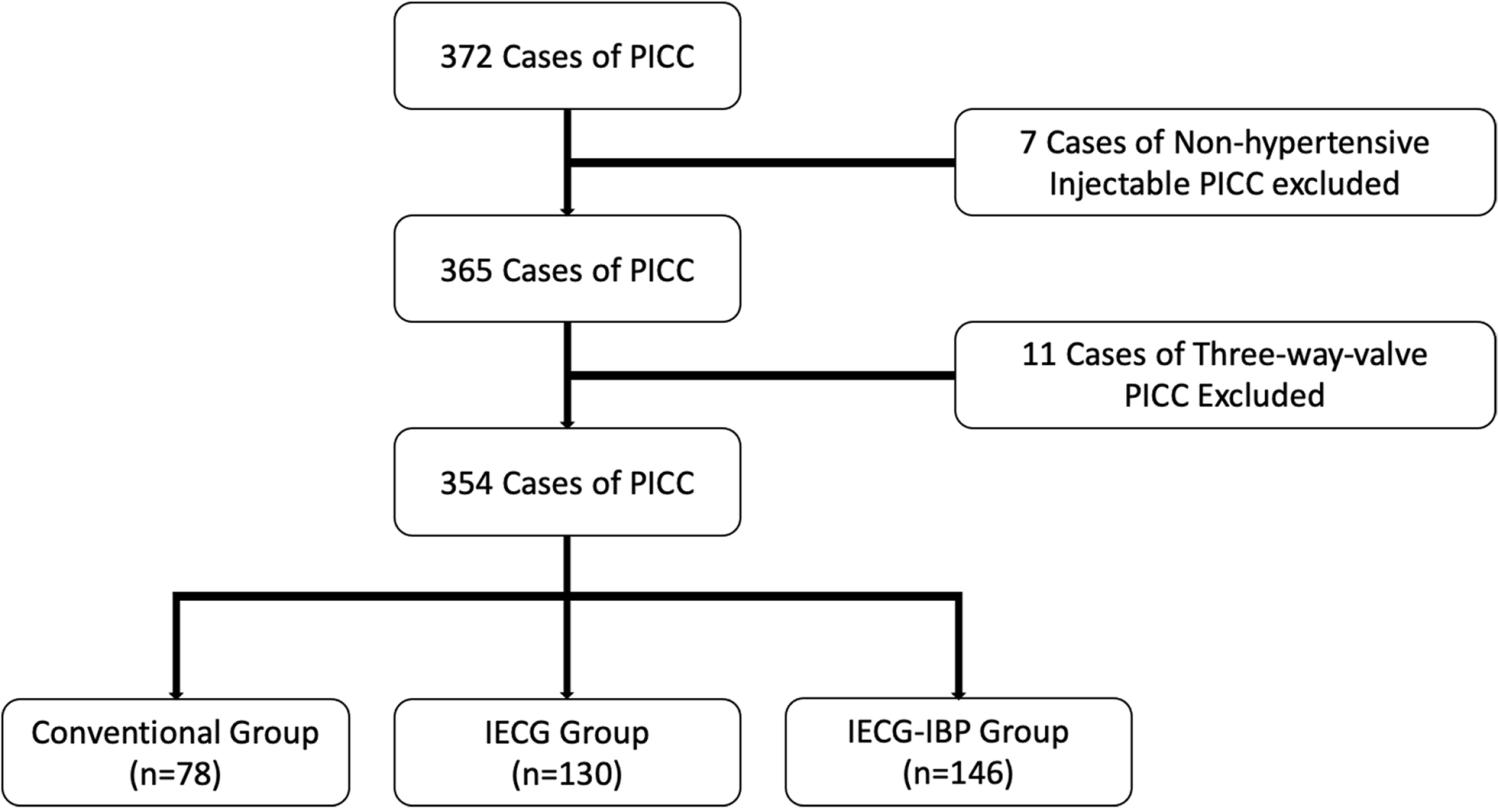
Flow diagram depicting the detailed selection of cases in this study

In this paper, a cohort of 372 patients who had peripherally implanted PICCs and were admitted to the Department of Neurosurgery at our hospital from April 2020 to April 2023 was studied. Clinical data such as age, gender, diagnosis, catheterized limb, vein, catheterization process, catheter material, etc., were recorded. The selection process of the study population is depicted as a flow chart in Fig. 1. Criteria for inclusion were applied as follows: the patient had to be 8-year old or older, and the intubation nurse had to possess PICC intubation qualification in neurosurgery. The utilized PICC was the 4Fr high-pressure resistant injection type with single cavity manufactured by Bard Medical Technology Co., Ltd. Cases that included non-high-pressure injection type single-chamber PICC, three-chamber valve PICC, or agitated patients were excluded. This study was approved by the Ethics Committee of Sun Yat-sen Memorial Hospital, Sun Yat-sen University, with the ethics review opinion number SYSKY-2022-362-01.

### Catheterization methods

#### Conventional group

**Fig. 2:**
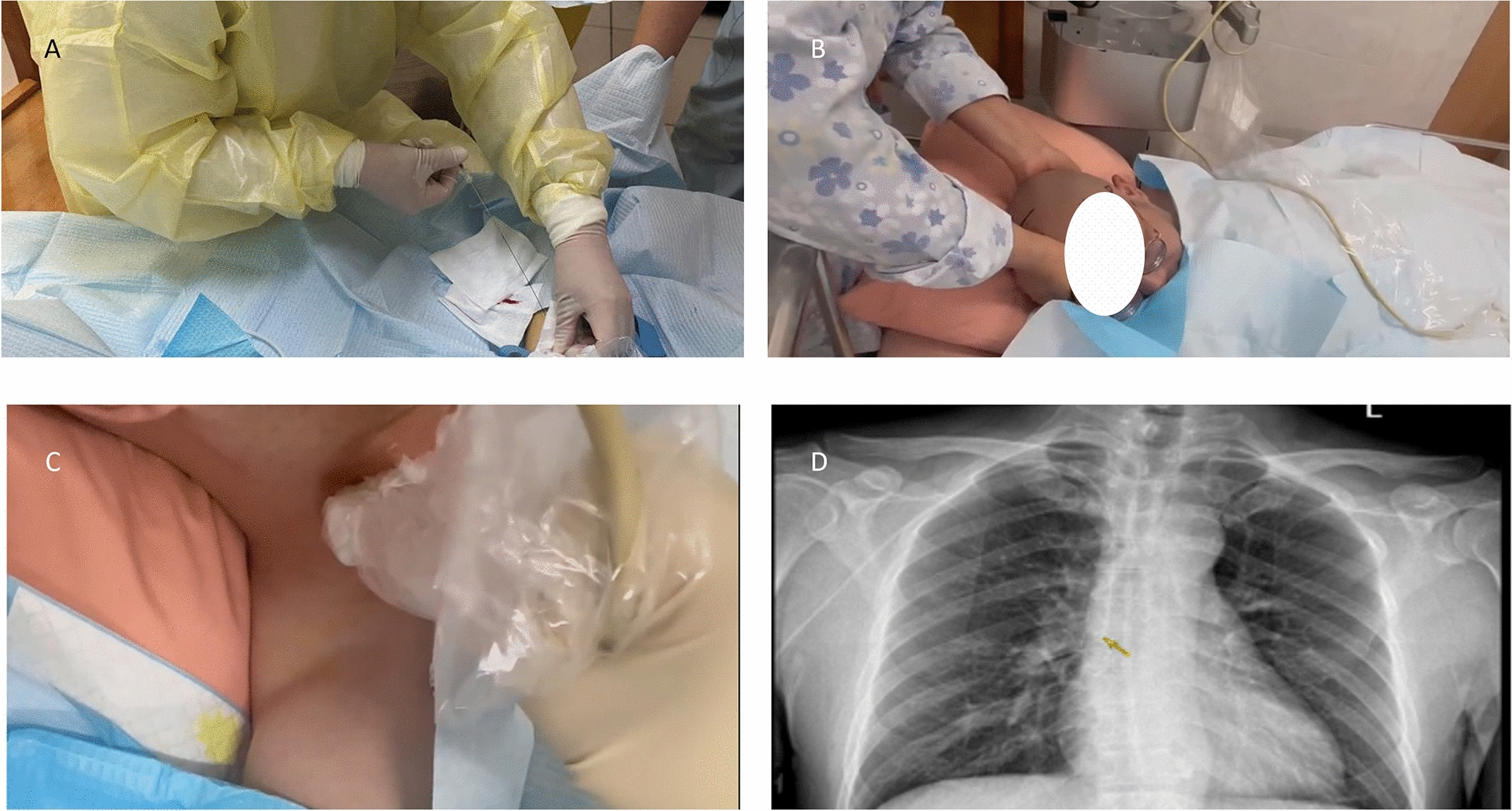
**A** Feeding guide wire. **B** Assistant helping the patient keep his chin close to his chest during delivery. **C** B-ultrasound examination of the blood vessels in the neck after insertion. D Infusion therapy after chest X-ray confirmation

The length of the catheter was measured, and the catheter was inserted following the standard procedure for PICC catheterization. Upon successful delivery, the position of the catheter tip was confirmed using chest X-ray checks. The procedure is shown in Fig. 2.

#### Intracavitary electrocardiographic (IECC) group

**Fig. 3:**
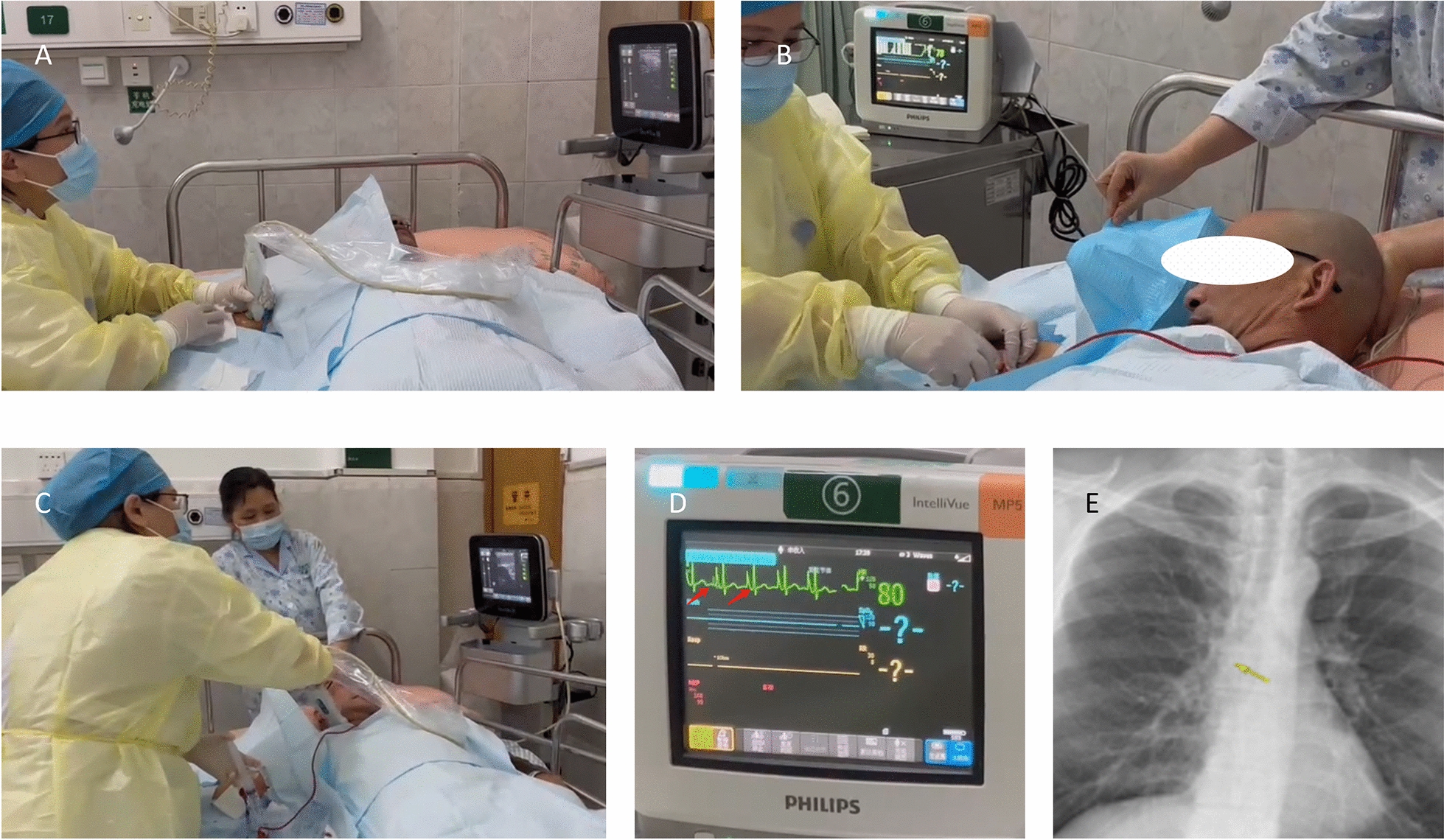
**A** Feeding guide wire. **B** Connecting the electrode wire, and the assistant helping the patient keep his chin close to his chest when the catheter is 15 cm into the catheter sheath. **C** B-ultrasound examination of the blood vessels in the neck after insertion. **D** Confirming the catheter insertion length with the specific P wave amplitude on the electrocardiogram. **E** Infusion therapy after chest X-ray confirmation

First, the patient was connected to the ECG monitor prior to the puncture operation, and it was adjusted to lead II. The basic waveform and heart rate of the patient's ECG were closely observed by the operator. When the catheter was advanced 15 cm into the introducer sheath, the alligator clip electrical connection wire was clamped to the PICC guide wire by the operator. The assistant then clamped the other end of the wire onto the RA lead wire of the monitor electrode wire. The catheter was then delivered by the operator following the conventional method, and the changes in P wave were observed on the electrocardiogram. After successful delivery, the position of the catheter tip was confirmed using chest X-ray checks. The procedure is shown in Fig. 3.

#### Intracavitary electrocardiographic with improved body positioning (IECG-IBP) group

**Fig. 4:**
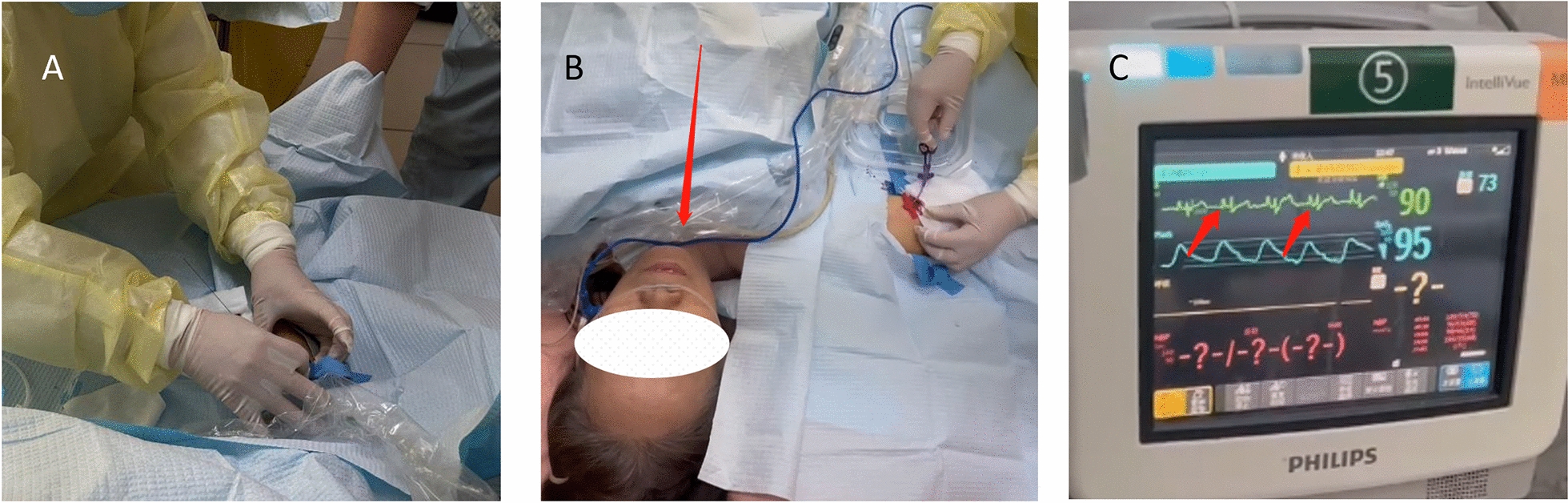
**A** Feeding guide wire. **B** Sending the catheter and applying electrode wires for intracavity electrocardiogram monitoring, while keeping the patient's head and neck in a centered position. **C** Confirming the catheter insertion length with the specific P wave amplitude on the electrocardiogram

The catheter was inserted based on the intracavity electrocardiographic positioning technique. Throughout the entire tube delivery process, the patient's head and neck were kept in a central position. When a specific P wave amplitude was observed in the patient's ECG, there was no need to conduct vascular ultrasonography to check the cervical vessels on the side of the patient's catheter. However, in cases where a specific P wave amplitude was not found, such ultrasonography was still necessary. The procedure is shown in Fig. 4.

### Evaluations

The indicators used for evaluation are the one-time success rate of catheter delivery and the catheter misplacement rate. Throughout the delivery process, the catheter was considered successfully delivered if it was never retracted. The placement of the catheter tip was assessed based on findings from chest X-rays. The optimal position for the catheter tip is in the lower third of the superior vena cava, near the junction of the superior vena cava and the right atrium [5, 7, 12, 15–17]. If the chest radiograph report shows that the catheter was located in the axillary vein, subclavian vein, brachiocephalic vein or internal jugular vein, it is considered misplaced.

### Statistical methods

The R software was used for statistical analysis. All analyzes were conducted using two-sided tests, with a confidence level of *α* = 0.05. Results with *P* < 0.05 were considered statistically significant. Quantitative data were described as “mean ± standard deviation” if normally distributed, and as median and interquartile range if not. Qualitative data were presented as frequency and percentage. *F* tests were employed for comparing groups based on the type of data.

## Results

Table 1 describes the gender, age, disease, catheterized limb, catheter puncture vein, intracranial midline structural shift, and cerebellum and brainstem compression of the three groups of patients.

**Table 1:** Comparative analysis of patient characteristics among three intubation groups

Term		Conventional group	IECG group	IECG-IBP group	F	*P*
Age		49.1 ± 17.2	50.0 ± 17.4	49.7 ± 17.5	0.080	0.923
Gender [*n*(%)]	Male	36(46.15)	55(42.31)	79(54.11)	1.994	0.138
	Female	42(53.85)	75(57.69)	67(45.89)	1.994	0.138
Intracranial tumor [*n*(%)]		47(60.26)	87(66.92)	85(58.22)	1.157	0.316
Intraspinal disease [*n*(%)]		3(3.85)	4(3.08)	4(2.74)	0.103	0.902
Subarachnoid hemorrhage [*n*(%)]		6(7.69)	6(4.62)	21(14.38)	4.098	0.017
Other types of cerebral hemorrhage [*n*(%)]		4(5.13)	11(8.46)	16(10.96)	1.090	0.337
Other cerebrovascular diseases [*n*(%)]		3(3.85)	11(8.46)	14(9.59)	1.192	0.305
Brain injury [*n*(%)]		3(3.85)	3(2.31)	6(4.11)	0.370	0.691
Other brain disorders [*n*(%)]		12(15.38)	8(6.15)	0(0)	12.010	0.000
Intracranial midline shift [*n*(%)]		11(14.10)	15(11.54)	33(22.60)	3.301	0.038
Cerebellum and brainstem compression [*n*(%)]		11(14.10)	14(10.77)	17(11.64)	0.263	0.769
Intracranial midline structures centered [*n*(%)]		56(71.79)	101(77.69)	96(65.75)	2.419	0.091
Left cannulation [*n*(%)]		15(19.23)	63(48.46)	75(51.31)	12.590	0.000
Right cannulation [n(%)]		63(80.77)	67(51.54)	71(48.63)	12.590	0.000
Basilic vein [*n*(%)]		73(93.59)	118(90.77)	126(86.30)	1.607	0.202
Brachial vein [*n*(%)]		5(6.41)	12(9.23)	20(13.70)	1.607	0.202

Table 2 presents the specific P wave amplitude changes during the catheterization of the three groups. In the conventional group, intracavity electrocardiography was not applied, and there were no specific P wave amplitude changes during catheterization. In the IECG group, 115 cases, or 88.46% of the total cases, showed changes in specific P wave amplitude. In the IECG-IBP group, there were 130 cases with changes in specific P wave amplitude, accounting for 91.78% of the cases. In addition, there was a case in the IECG-IBP group where misplacement of the catheter into contralateral internal jugular vein occurred, and no specific P wave amplitude change was observed.

**Table 2:** P Wave characteristics in three patient groups during intubation [*n*(%)]

Group	Count	Changed P	Inverted P	Unchanged *P* Increased R	Unchanged *P*
Conventional group	78	0(0)	0(0)	0(0)	0(0)
IECG group	130	115(88.46)	0(0)	4(3.08)	11(8.46)
IECG-IBP group	146	134(91.78)	2(1.37)	0(0)	10(6.85)

Table 3 documents the one-time success rate of catheter delivery for the three groups. Table 4 records the locations of the PICC tips. Internal jugular vein ectopia is defined as catheter tip ectopia by observing the neck blood vessels on the side of the catheter after the first successful catheter delivery, or by chest X-ray examination showing that the catheter is located in the internal jugular vein. This project aims to test an improvement on the traditional PICC catheterization operation process. In Table 3, there is no statistical significance in the success rate of one-time tube delivery for the three groups of patients, proving that process improvement will not increase the failure rate of one-time tube delivery. With the conventional method, the patient's head turns to the side of the tube and the lower jaw is close to the chest. After successful catheter delivery, ultrasound exploration of the internal jugular vein is performed to prevent catheter malposition. We conducted a simplified study on the conventional process and found that there was no statistical significance in the catheter tip placement rate between the IECG-IBP group and the conventional group, as shown in Table 4. Patients in the IECG-IBP group did not experience an increased incidence of catheter internal jugular vein malposition due to changes in the catheter delivery process.

**Table 3:** Comparison of one-time success rates for catheter delivery [*n*(%)]

Group	Count	One-time successful delivery	
		Yes	No
Conventional group	78	72(92.31)	6(7.69)
IECG group	130	125(96.15)	5(3.84)
IECG-IBP group	146	138(94.52)	8(5.37)
F-statistic		0.710	0.710
*p*-value		0.492	0.492

**Table 4:** Comparison of PICC placement rates and abnormal positions [*n*(%)]

Group	Count	Tip in place	Tip misplacement		
			Internal jugular vein	Beyond the superior vena cava	Upper segment of superior vena cava
Conventional group	78	69(88.46)	4(5.13)	2(2.56)	3(3.85)
IECG group	130	123(94.62)	5(3.85)	1(0.77)	1(0.77)
IECG-IBP group	146	138(94.52)	7(4.79)	0(0)	1(0.68)
F-statistic		1.796	0.114	2.002	2.137
*p*-value		0.168	0.893	0.137	0.120

Among the three groups of patients, there were 10 patients in the conventional group, accounting for 12.82%, and 17 patients in the IECG group, accounting for 13.08%. All these patients had neck pain during PICC catheterization and resisted moving their jaw closer to their chest. There were 25 people in the IECG-IBP group, accounting for 17.12%, and none of them had the above symptoms. There were 15 patients with original symptoms of headache and dizziness in the conventional group, accounting for 19.23%; 16 patients in the IECG group, accounting for 12.31%. The original symptoms of these patients were aggravated during the PICC catheterization process. There were 45 people in the IECG-IBP group with such symptoms, accounting for 30.82%; three of these patients had ectopic catheters in the internal jugular veins, and the symptoms above were aggravated by changing the head position and adjusting the catheter. The results are shown in Table 5.

**Table 5:** Symptoms of the three groups of patients during catheterization

Group	Counts	Neck pain and resistance	Aggravated headache and dizziness
Conventional Group	78	10 (12.82)	15 (19.23)
IECG Group	130	17 (13.08)	16 (12.31)
IECG-IBP Group	146	0 (0)	3 (2.05)
*F*-statistics		10.800	9.956
*p* value		0.000	0.000

## Discussion

### The impact of intracavitary ECG-guided PICC tip positioning technology on PICC catheterization outcomes in neurosurgery patients

Our study found that the proportion of specific P wave amplitude changes was 88.46% in the IECG group and 91.78% in the IECG-IBP group, which is consistent with rates reported in previous literature of 91.04% or higher [9, 15, 18]. Within the IECG group, among patients with no specific P wave amplitude changes, 2 had the PICC tip located at or above the upper segment of the superior vena cava, 3 had the tip at the right atrium, and 1 exhibited arrhythmia. In the IECG-IBP group, among patients with no specific P wave amplitude changes, 2 had the tip located at or above the upper segment of the superior vena cava, and 1 had it in the contralateral internal jugular vein. One case involved an inverted P wave in a patient with double superior vena cava. During catheter insertion, the patient’s heart rate increased significantly, prompting the nurse to retract the catheter, which resulted in a change in the inverted P wave. The patient’s heart rate returned to normal when catheterization was terminated, and a chest X-ray confirmed that the PICC tip was located in the inferior segment of the superior vena cava. In this study, no cases of specific P wave changes were observed due to the location of the catheter tip at or above the upper segment of the superior vena cava or arrhythmia.

Given that most neurosurgery patients do not cooperate well during the procedure and are often in poor condition, it is recommended to trim the length of the catheter after successful PICC puncture for open-ended catheters to prevent them from being located at or above the superior vena cava. In cases of specific P wave regression, bidirectional P waves, or negative P waves, the catheter should be withdrawn until a positive P wave is observed or the specific P wave amplitude is approximately half- to three-quarters of the R wave amplitude. At this point, the length of PICC insertion is considered ideal [9–11, 19–22]. In this study, high-pressure injection-resistant PICCs were used, and the catheter guide wire was trimmed to be 1 cm shorter than the catheter.

Moreover, the specific P wave amplitude is closely related to the patient’s body shape. For tall and thin patients, the catheter should be withdrawn by 0.5 cm when the specific P wave reaches its highest amplitude. In obese patients, the catheter tip position is considered ideal when the catheter is withdrawn by 1 cm as specific P wave reaches its highest value. As shown in Table 1, this study included patients who primarily experienced symptoms such as dizziness, headache, disturbance of consciousness, cognitive dysfunction, restlessness, or had indwelling tracheostomy tubes, among others. The assistance of one or more skilled assistants was required during the indwelling PICC procedure. The application of PICC tip positioning technology guided by intracavity electrocardiographic monitoring proved valuable in detecting abnormal catheter tip positions during the tube delivery process, improving the success rate of catheter insertion, as presented in Table 3. This technique also reduced the risk of PICC contamination and saved operating time, particularly in cases involving post-tracheotomy patients and those with impaired consciousness.

### The impact of combined intracavitary ECG-guided PICC tip positioning technology and improved body positioning on PICC catheterization outcomes in neurosurgery patients

In the 2016 version of the practice standard recommendation, the safest location for placement of the PICC tip in both adults and children is at the junction of the superior vena cava and right atrium. The most common misplacement of the PICC occurs in the internal jugular vein, with an incidence rate ranging from 3.31 to 12.30%, while the internal jugular vein accounts for 98% of cases of catheter malposition [23, 24]. In the conventional PICC tube insertion process, the practitioner needs to turn the patient’s head to the side where the tube is inserted so that the chin is close to the chest. The positional change aims to reduce the incidence of internal jugular vein ectopy during catheterization. In our study, the rate of catheter misplacement in the internal jugular vein was 4.79% in the IECG-IBP group, which was lower than the rate of 5.13% in the conventional group. Within the IECG-IBP group, internal jugular vein misplacement occurred in seven cases. In five cases, the catheter was immediately retracted and reintroduced, resulting in specific P wave changes detected on the ECG, and the catheter tip was confirmed to be in an ideal position based on the chest X-ray results. In one case, there was no specific P-wave change during re-catheterization, but the position of the catheter tip was still ideal. In one patient, the catheter was successfully reintroduced, but it was misplaced in the contralateral internal jugular vein. Based on our findings, there is no statistically significant evidence suggesting an increased incidence of internal jugular vein misplacement due to this improved catheter delivery method.

Glioma symptoms primarily result from tumor mass effects. High-grade gliomas often cause acute increases in intracranial pressure, disturbances of consciousness, unstable vital signs and brain herniation [25]. The severity of these symptoms tends to correlate with the tumor grade. During PICC catheterization, a patient with frontal lobe and parietal lobe space-occupying lesions experienced head position changes and vomited. In a case of thalamic glioma with the catheter tip abnormally located in the internal jugular vein, the catheter required two adjustments, and the patient experienced severe dizziness and cyanosis when the head position was changed. Even after the third catheter adjustment, it remained misplaced in the contralateral internal jugular vein. Finally, the PICC was successfully delivered under X-ray guidance with the cooperation of two practitioners. Due to the limited volume of the posterior fossa and increased intracranial pressure caused by the tumor, large tumors are prone to sudden foramen magnum herniation, especially in cases with significant preoperative increases in intracranial pressure [26]. For patients who have undergone tracheotomy, changes in head position can easily lead to irritating coughs, increasing the risk of sputum droplet contamination of the PICC. Patients with subarachnoid hemorrhage or other types of intracerebral hemorrhage may experience aggravated pain with changes in head and neck positions. In particular, the severity of symptoms tends to correlate with the Hunt-Hess scale. In severe cases, this may cause acute intracranial hypertension or induce seizures [27, 28]. Severe patient discomfort can also cause vasoconstriction, further complicating the delivery of the tube. Our study found that in the conventional group and IECG group, patients with nuchal ankylosis and tracheostomy accounted for 12.82% and 13.08% respectively, and in the IECG-IBP group, patients with nuchal ankylosis and tracheostomy accounted for 17.12%. Both the conventional group and the intracavitary electrocardiographic positioning group experienced neck pain and resistance (100%), which was significantly higher than that in the IECG-IBP group (0%). In addition, patients with original dizziness and headache symptoms accounted for 19.23% and 12.31% in the conventional group and IECG group, and 30.82% in the IECG-IBP group. The proportion of patients with worsening symptoms in the conventional group and IECG group (100%) was significantly higher than that in the IECG-IBP group (0%). Since many neurosurgery patients suffer from symptoms such as tracheotomy, neck stiffness, dizziness and headache, our study shows that the IECG-IBP tube delivery method is more beneficial to neurosurgery patients and can effectively reduce irritation to these patients. Patient intracranial pressure monitoring can be achieved through intracranial Doppler, retinal reflection, sensor placement, lumbar puncture, etc. For PICC procedures, Doppler and retinal reflection are more feasible. In subsequent studies, we will add these methods to evaluate the changes in intracranial pressure during PICC and guide the completion of PICC more safely and effectively.

We do acknowledge the limitations of this study. Due to the sample size limitations of this project, there may be some biases present. However, during the catheterization of hyperbaric injectable PICCs in neurosurgical patients, the application of intracavitary ECG-guided PICC tip positioning technology combined with improved body positioning provides more benefits and improves patient comfort.

## Data Availability

All data generated or analyzed during this study are included in this published article.
